# *KRAS* or *BRAF* mutation status is a useful predictor of sensitivity to MEK inhibition in ovarian cancer

**DOI:** 10.1038/sj.bjc.6604783

**Published:** 2008-11-18

**Authors:** N Nakayama, K Nakayama, S Yeasmin, M Ishibashi, A Katagiri, K Iida, M Fukumoto, K Miyazaki

**Affiliations:** 1 Departments of Obstetrics and Gynecology, Shimane University School of Medicine, Izumo, Japan; 2 Departments of Pathology, Institute of Development, Aging and Cancer, Tohoku University, Sendai, Japan

**Keywords:** ovarian carcinoma, *KRAS*, *BRAF*, mutation, ERK1/2, CI-1040

## Abstract

This study examined the status of *KRAS* and *BRAF* mutations, in relation to extracellular signal-regulated protein kinase (ERK) activation in 58 ovarian carcinomas to clarify the clinicopathological and prognostic significance of *KRAS/BRAF* mutations. Somatic mutations of either *KRAS* or *BRAF* were identified in 12 (20.6%) out of 58 ovarian carcinomas. The frequency of *KRAS/BRAF* mutations in conventional serous high-grade carcinomas (4.0% : 1/25) was significantly lower than that in the other histological type (32.3% : 10/31). Phosphorylated ERK1/2 (p-ERK1/2) expression was identified in 18 (38.2%) out of 45 ovarian carcinomas. *KRAS/BRAF* mutation was significantly correlated with International Federation of Gynecology and Obstetrics (FIGO) stage I, II (*P*<0.001), and p-ERK1/2 (*P*<0.001). No significant correlations between *KRAS/BRAF* mutations or p-ERK1/2 expression and overall survival were found in patients with ovarian carcinoma treated with platinum and taxane chemotherapy (*P*=0.2460, *P*=0.9339, respectively). Next, to clarify the roles of ERK1/2 activation in ovarian cancers harbouring *KRAS* or *BRAF* mutations, we inactivated ERK1/2 in ovarian cancer cells using CI-1040. Cl-1040 is a compound that selectively inhibits MAP kinase kinase (MEK), an upstream regulator of ERK1/2, and thus prevents ERK1/2 activation. Profound growth inhibition and apoptosis were observed in CI-1040-treated cancer cells with mutations in either *KRAS* or *BRAF* in comparison with the ovarian cancer cells containing wild-type sequences. This was evident in both *in vitro* and *in vivo* studies. The findings in this study indicate that an activated ERK1/2 pathway is critical to tumour growth and survival of ovarian cancers with *KRAS* or *BRAF* mutations. Furthermore, they suggest that the CI-1040-induced phenotypes depend on the mutational status of *KRAS* and *BRAF* in ovarian cancers. Therefore, ovarian cancer patients with *KRAS* or *BRAF* mutations may benefit from CI-1040 treatment.

Ovarian carcinoma is the most lethal malignant disease in American women (Wingo *et al*, 1995) and the most lethal gynaecological cancer in Japan. Its frequency has increased dramatically in the last decade. In more than 70% of patients with ovarian carcinoma, there is evidence of tumour dissemination beyond the ovaries at diagnosis. In these cases, combined treatment with surgery and chemotherapy is necessary. First-line chemotherapy with platinum drugs and taxanes yields a response rate of over 80%, but almost all patients relapse. Although there are well-established surgical and chemotherapeutic treatments for ovarian cancer, there is a significant opportunity to develop drugs targeting specific molecular pathways. Drugs of this type would be particularly useful for recurrent disease that has acquired chemoresistance. Thus, there is a need for an improved understanding of the molecular pathways of ovarian carcinogenesis. Several genetic alterations are associated with ovarian carcinogenesis. The most frequent genetic abnormalities in ovarian carcinoma are mutations in *KRAS*, *BRAF,* and *p53* (Singer *et al*, 2003; Nakayama *et al*, 2006).

Mutations of either *BRAF* or *KRA*S lead to constitutive activation (phosphorylation) of their downstream target, mitogen-activated protein kinase (MAPK), also known as extracellular signal-regulated protein kinase (ERK) (Olson and Hallahan, 2004; Wan *et al*, 2004). Mutations in *BRAF* or *KRAS* are correlated with overexpression of activated ERK1/2 in ovarian serous tumours (Hsu *et al*, 2004). Activation of ERK1/2 in turn activates downstream cellular targets (Peyssonnaux and Eychene, 2001; Allen *et al*, 2003) including a variety of cellular and nuclear proteins. Although the functions of the RAS–RAF–MEK–ERK pathway and its downstream effectors have been recently explored, only the serous type of ovarian cancer has been studied (Hsu *et al*, 2004; Pohl *et al*, 2005). In addition, the biological role of this pathway in the development of ovarian cancers of other histological types is unknown.

Activating *KRAS* and *BRAF* mutations typically show mutant exclusivity in tumours (Brose *et al*, 2002; Davies *et al*, 2002; Gorden *et al*, 2003; Singer *et al*, 2003).

A large proportion of microsatellite-stable colorectal tumour metastases has been shown to accumulate *BRAF*/*KRAS* mutations (Oliveira *et al*, 2007). This suggests an epistatic relationship in which either mutation is sufficient to deregulate a common effector pathway, such as the MAP kinase kinase (MEK)–ERK kinase cascade. If this is the case, tumours arising as a result of a mutation in either *KRAS* or *BRAF* should harbour similar downstream dependencies. These might represent useful therapeutic targets in ovarian cancer. To test this hypothesis, we examined the consequences of MEK–ERK pathway inhibition using a highly potent and selective inhibitor of MEK1/2, CI-1040 (formerly known as PD184352) (Schaeffer and Weber, 1999; Sebolt-Leopold *et al*, 1999, 2003; Sebolt-Leopold, 2004). The inhibitor was tested in a collection of ovarian cancer cell lines that showed differing mechanisms of MAP kinase pathway deregulation.

## Materials and methods

### Tissue samples

Formalin-fixed, paraffin-embedded tissue samples of 58 ovarian cancers, including 27 serous carcinomas, 20 mucinous carcinomas, and 11 endometrioid carcinomas were used in this study. These samples were obtained from the Department of Obstetrics and Gynecology at the Shimane University Hospital. Diagnosis was based on conventional morphological examination of sections stained with haematoxylin and eosin (H&E) staining, and tumours were classified according to the WHO (World Health Organization) classification. Tumour staging was carried out according to the International Federation of Gynecology and Obstetrics (FIGO) classification. The clinicopathological characteristics of the patients included in this study are summarised in Table 1. All the patients were primarily treated with cytoreductive surgery and adjuvant platinum and taxane chemotherapy (CBDCA AUC5, Paclitaxel 175 mg m^−2^ or Docetaxel 70 mg m^−2^). All the cases received 6–12 courses of this regimen. The acquisition of tumour tissues was approved by the Shimane University Institutional Review Board. The paraffin tissue blocks were organised into tissue microarrays, which were made by removing 3 mm diameter cores of tumour from each block. The areas for coring were selected by surgical pathologists (MF) on the basis of a review of the H&E slides.

### Cell culture and cell lines

OVCAR3, SKOV3, A2780, MDAH2774 (serous carcinoma), and ES2 (clear cell carcinoma) human ovarian cancer cell lines were obtained from the American Tissue Culture Center (Rockville, MD, USA). The human ovarian carcinoma cell line KF28 (serous carcinoma) was a kind gift from Dr Yoshihiro Kikuchi (Ohki Memorial Kikuchi Cancer Clinic for Women, Saitama, Japan) (Yamamoto *et al*, 2000). The MPSC1 cell line was established from a low-grade serous carcinoma and was a kind gift from Dr le-Ming Shih (Johns Hopkins Medical Institutions, Baltimore, MD, USA). OVK#18 (serous carcinoma) human ovarian cancer cell line was obtained from Tohoku University (Sendai, Japan). OMC3 (mucinous carcinoma) and JHOC5 (clear cell carcinoma) human ovarian cancer cell lines were also obtained from Riken Bioresource Center (Ibaragi, Japan). In addition, human papillomavirus E6/E7-immortalised primary cultures of normal ovarian surface epithelium (OSE) were also included in this study. IOSE27, a normal OSE cell line, was obtained from the American Tissue Culture Center. OSE7 and OSE10 normal OSE cell lines were a kind gift from Dr Hidetaka Katabuchi (Kumamoto University, Kumamoto, Japan).

A set of primary cultures was established from ovarian cancers, including POC-1, POC-2, and POC-3. The acquisition of anonymous tissue specimens was approved by the Shimane University Institutional Review Board.

The diagnoses were confirmed by a surgical pathologist before the tumour samples were harvested for experiments. Primary tumour cultures were established from freshly isolated tumour samples by immunosorting or trypsinisation. For immunosorting, fresh tumour tissues were minced and incubated with collagenase A (2 mg ml^−1^) at 37 °C for 40 min. After filtration through sieve membranes (with 100 *μ*m pores), tumour cells were immunosorted using an epithelial specific antigen (Ep-CAM) antibody bound to Dynal™ beads (Dynal, Oslo, Norway) following the vendor's instructions (Nakayama *et al*, 2006). Freshly isolated tumour cells were allowed to grow in culture and were used for experiments within two passages.

### Mutational analysis of *KRAS* and *BRAF*

Genomic DNA was purified from all the cell lines and formalin-fixed, paraffin-embedded tissues using a Qiaquick polymerase chain reaction (PCR) purification kit (Qiagen, Valencia, CA, USA). PCR was then carried out followed by nucleotide sequencing using the iCycler (Bio-Rad, Hercules, CA, USA). Exon 1 of *KRAS* and exon 15 of *BRAF* were both sequenced, as these mutational hot spots together harbour nearly all published mutations (Davies *et al*, 2002; Singer *et al*, 2002, 2003; Sieben *et al*, 2004). The primers for PCR and sequencing were manufactured by GeneLink (Hawthorne, NY, USA), and their sequences were described in an earlier report (Nakayama *et al*, 2006). The sequences were analysed using the Lasergene programme, DNASTAR (Madison, WI, USA).

### Immunohistochemistry

Expression of the active phosphorylated ERK1/2 (p-ERK1/2) was assessed by immunohistochemistry and western blot analysis. The antibody used in this study was a rabbit polyclonal antibody that reacted with phosphorylated but not unphosphorylated ERK1/2 (Cell Signaling Technology). Immunohistochemistry was carried out on tissue microarrays at a dilution of 1 : 1000 followed by detection with the En Vision+ System using the peroxidase method (DAKO, Carpinteria, CA, USA). The percentage of positive cells was estimated by randomly counting ∼500 tumour cells from three different high-power fields (× 40) within one specimen. A positive reaction was defined as a discrete localisation of the brown chromagen in the nucleus or cytoplasm. Cases in which more than 5% of the tumour cells showed detectable immunoreactivity were scored as positive.

### Western blot analysis

Cell lysates were prepared by dissolving cell pellets in Laemmli sample buffer (BioRad, Hercules, CA, USA) supplemented with 5% *β*-mercaptoethanol (Sigma, St Louis, MO, USA). Western blot analysis was performed on ovarian cancer/OSE cell lines/cultures, including OVCAR3, SKOV3, A2780, MDAH2774, ES2, MPSC1, KF28, OVK#18, OMC3, JHOC5, PC1, PC2, PC3, IOSE29, OSE7, and OSE10. Similar amounts of total protein from each lysate were loaded and separated on 10% Tris-Glycine-SDS polyacrylamide gels (Novex, San Diego, CA, USA) and electroblotted to Millipore Immobilon-P polyvinylidene difluoride membranes. The membranes were probed with an active ERK1/2 antibody (pTEpY, 1 : 5000) (Cell Signaling Technology) followed by a peroxidase-conjugated anti-mouse or anti-rabbit immunoglobulin (1 : 20 000). The same membrane was probed with an antibody that reacted with total ERK1/2 (1 : 5000) (Cell Signaling Technology) for loading controls. Western blots were developed by chemiluminescence (Pierce, Rockford, IL, USA).

### Cell-growth assays

For the cell-growth assay, cells were plated at the same density (3 × 10^3^ cells per well) in 96-well plates. An methyl thiazoyl tetrazorium (MTT) cell-growth assay was performed (Nakayama *et al*, 2001) 96 h after treating the cells with CI-1040 (provided by Pfizer, Inc., New York, NY, USA) at 5 *μ*M or with dimethyl sulphoxide (DMSO) (control). The data were expressed as a percentage of the DMSO control. The mean and standard deviation (s.d.) were obtained from three experiments. Apoptotic cells were detected with 4',6-diamidino-2-phenylindole (DAPI) staining. The data were expressed as the mean ±1 s.d. from triplicates. To confirm the presence of apoptotic cells, DAPI-stained cells were also stained with Annexin V dye. Bromodeoxyuridine (BrdUrd) uptake and staining were measured using a cell proliferation kit (Amersham, Buckinghamshire, England, UK) and apoptotic cells were detected using an Annexin V staining kit (Bio Vision, Mountain View, CA, USA). The percentages of BrdUrd-positive and Annexin V-positive cells were determined by counting approximately 400 cells from each well in 96-well plates. The data were expressed as the mean ±1 s.d. of triplicates.

### Tumour xenograft in nude mice

To confirm the findings of a CI-1040 effect *in vitro*, we injected 3 × 10^6^ MDAH2774 or SKOV3 cells into the intraperitoneal tissue of *nu/nu* mice (4 weeks of age). Four weeks BALB/c *nu/nu* mice were purchased from Charles River Japan Inc. (Kanagawa, Japan). CI-1040 was prepared in a vehicle of 10% Cremophore EL (Sigma, St Louis, MO, USA), 10% ethanol, and 80% water. When the model for the mouse study was first designed, the end point was set to be the day when the mice began to produce ascites, or acute gain of weight due to tumour growth, for reasons of ethical origin. Tumours that start causing ascites have a chance of developing other malignant characteristics, which can become harmful progressively. Four mice were used for each experimental group. During the study, the mice were killed when it was discovered that the abdomen of one of the mice had begun to distend because of ascites. One week after tumour-cell injection, either CI-1040 (CI-1040, 150 mg kg^−1^, resuspended in 10% Cremophore EL (Sigma), 10% ethanol, and 80% water) or a vehicle only (10% Cremophore EL (Sigma), 10% ethanol, and 80% water) were injected intraperitoneally (i.p.) once daily for 3 weeks. The total dose of CI-1040 for each mouse was 63 mg. Four weeks after the cell injection (three weeks after CI-1040 injection), the abdomens of the control group mice had begun to distend. The time for termination of the experiment was dictated by the aforementioned ethical reasons (tumour ascites in controls), and this endpoint was observed at that time. We anaesthetised the mice before they were rendered moribund by the experiment. The total tumour weight at that time was around 500 mg. Necropsy was carried out on all mice to assess i.p. tumour growth, and the tumours were excised and weighed. Animal experiments were carried out in accordance with the regulations of the Institutional Ethical Commission (Shimane University) and of the United Kingdom Co-ordinating Committee on Cancer Research guidelines (Workman, 1998).

### Statistical methods for clinical correlation

Overall survival was calculated from the date of diagnosis to the date of death or last follow-up. Patients with either *KRAS* or *BRAF* mutations had similar performance status distributions. The data were plotted as Kaplan–Meier curves, and the statistical significance was determined by the log-rank test. Data were censored when patients were lost to follow-up. The Student's *t*-test was used to examine the statistical significance in the difference of growth-assay data.

## Results

### Identification of *KRAS* and *BRAF* mutations

The mutational status of *KRAS* and *BRAF* in all 45 ovarian carcinomas is summarised in Table 1. Somatic mutations of *KRAS* were identified in 8 (13.7%) out of 58 ovarian carcinomas. In contrast, somatic mutations of *BRAF* were identified in 5 (8.6%) out of 58 ovarian carcinomas. Somatic mutations of either *KRAS* or *BRAF* were identified in 12 (20.6%) out of 58 ovarian carcinomas. Most *KRAS* mutations were located at codon 12 and all *BRAF* mutations at codon 600. Both of these codons are mutation hot spots. Interestingly, simultaneous mutations of *KRAS* and *BRAF* did not occur in the tested ovarian carcinomas with the exception of one mucinous case.

**Figure 1:**
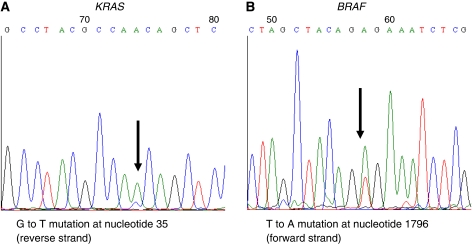
Chromatograms of *KRAS* and *BRAF* mutational status in three representative ovarian cancer cells. (**A**) Left panel (MDAH2774) showing a point mutation in the *KRAS* gene. (**B**) Right panel (ES2) showing a point mutation in the *BRAF* gene. Arrows represent spike which indicates mutation.

A panel of ovarian cancer cell lines and primary cultures was first analysed for tumour mutation status in the *KRAS* and *BRAF* genes. As shown in Figure 1, three ovarian cancer cell lines harboured either *KRAS* or *BRAF* mutations. The frequency of either *KRAS* or *BRAF* mutations in conventional serous high-grade carcinomas (4.0% : 1/25) was significantly lower than in the other histological type (32.2% : 10/31).

### Relationship between *KRAS/BRAF* mutations and p-ERK1/2 expression or clinicopathological factors

**Figure 2:**
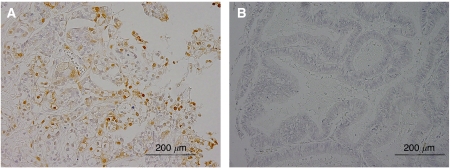
Immunohistochemical staining of phosphorylated extracellular-regulated kinase (p-ERK1/2). (**A**) Intense immunoreactivity is present in both the nucleus and the cytoplasm in this ovarian carcinoma. (**B**) A case with negative staining of phosphorylated ERK1/2 (p-ERK1/2).

The immunoreactivity of active p-ERK1/2 was detected in both the nucleus and the cytoplasm of the tumour cells (Figure 2). This is consistent with an earlier report (Mizumoto *et al*, 2007). Positive active p-ERK1/2 was identified in 27 (46.6%) out of 58 ovarian carcinomas. The patients were stratified into two groups depending on the mutational status of *KRAS/BRAF*. The relationships between *KRAS/BRAF* mutations and clinicopathological factors, including p-ERK1/2 expression are shown in Table 2. There was no significant correlation between *KRAS/BRAF* mutations and the patient's age. The results in Table 2 show that *KRAS/BRAF* mutation is correlated significantly with FIGO stage I, II (*P*<0.001), and p-ERK1/2 (*P*<0.001). In addition, there were significant correlations between *KRAS/BRA*F mutations and pathological grade (*P*=0.004), and histological subtype (*P*=0.014).

### Effect of *KRAS/BRAF* mutations or p-ERK1/2 on the prognosis of ovarian carcinomas

**Figure 3:**
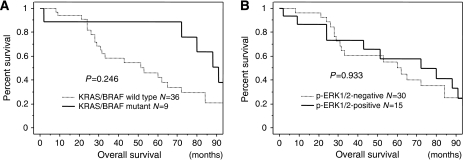
Kaplan–Meier survival curve in 45 patients with ovarian carcinoma according to *KRAS/BRAF* mutation and phosphorylated ERK (p-ERK) expression. (**A**) KRAS/BRAF mutational status correlates with favourable overall survival in patients with ovarian carcinoma. (**B**) p-ERK1/2 expression does not correlate with shorter overall survival in patients with ovarian carcinoma.

Next, we examined the prognostic effect of *KRAS/BRAF* mutations and p-ERK1/2 expression. Out of the 58 samples that we examined, 45 were available for prognostic analysis. Kaplan–Meier estimates of overall survival are plotted in Figure 3. There was no significant relationship between *KRAS/BRAF* mutations or p-ERK1/2 expression and overall survival in patients with ovarian carcinoma (*P*=0.2460, *P*=0.9339, respectively). Univariate analysis showed that only FIGO stage III, IV affected the overall survival of patients with ovarian carcinoma significantly(*P*=0.014).

### Effects of ERK1/2 inactivation on ovarian carcinoma *in vitro*

**Figure 4:**
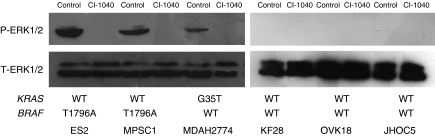
Western blot analysis. Expression of phosphorylated ERK1/2 (p-ERK1/2) is undetectable in all CI-1040-treated samples. A similar amount of protein was loaded in CI-1040 and DMSO-treated samples as evidenced by a similar intensity of total ERK1/2. D, DMSO treatment, C, and CI-1040 treatment.

**Figure 5:**
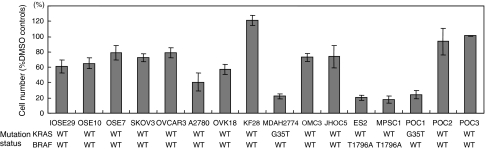
Effects of CI-1040 on cell proliferation. Cells were counted after 72 h of CI-1040 or DMSO (control) treatment. The mutational status of *KRAS* and *BRAF* for each sample is shown under the cell lines and primary cancer cell cultures. Ovarian cancers with mutations in either *KRAS* or *BRAF* are more sensitive to growth inhibition by CI-1040 than those with wild-type (WT) sequences.

A panel of ovarian cancer cell lines and primary cultures of ovarian cancer were first analysed for *KRAS* and *BRAF* gene mutation status. Mutational status was correlated with growth inhibition and apoptosis induction by the MEK inhibitor CI-1040 that prevented activation of the downstream target, ERK1/2. Western blot analysis showed a dose-dependent effect on the expression of active ERK1/2 in ES2 cells, and active ERK1/2 was not detectable 6 h after treating the cells with CI-1040 at a concentration of 5 *μ*M (Figure 4). As shown in Figure 5, four of the tumours harbouring either *KRAS* or *BRAF* mutations showed a marked reduction (<50% of DMSO control) in the cell number in the CI-1040-treated group as compared with the other 14 tumours containing wild-type *KRAS* and *BRAF* (*P*<0.001). CI-1040 had no significant effect on the growth of normal cells, including the OSE cells. It is likely that *KRAS/BRAF* mutation is not the only determinant for activating ERK1/2. Therefore, we analysed p-ERK1/2 expression in each of the cell lines listed in Figure 5. Only four of these cell lines, MDAH2774, ES2, MPSC1, and POC1, strongly expressed p-ERK1/2. SKOV3 and A2780 showed weak expression of ERK1/2.These results suggest that activation of ERK1/2 may depend on *KRAS/BRAF* mutation in ovarian cancer cells.

**Figure 6:**
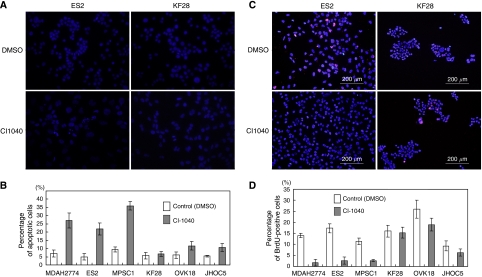
Detection of apoptotic cells and proliferation cells. (**A**) The CI-1040-treated ES2 cells, but not KF28 cells, show morphological features typical of apoptosis. (**B**) Apoptotic cells are quantified by counting them under a fluorescent microscope. (**C**) Treatment with CI-1040 decreases DNA synthesis as measured by BrdUrd uptake in ES2 cells, but not KF28 cells. (**D**) Proliferation is estimated by counting BrdUrd-stained cells under a fluorescent microscope. The experiment was performed 72 h after CI-1040 or DMSO treatment.

To assess the mechanisms underlying growth inhibition by CI-1040, we measured the percentages of BrdUrd-labelled cells and Annexin V-labelled cells to estimate proliferation and apoptosis, respectively. We found that CI-1040 significantly reduced cellular proliferation and induced apoptosis in cell lines with either *KRAS* or *BRAF* mutations in comparison with cell lines with wild-type sequences (Figure 6, Supplementary Figure 1).

### Effects of CI-1040 ERK1/2 inactivation on ovarian carcinomas *in vivo*

**Figure 7:**
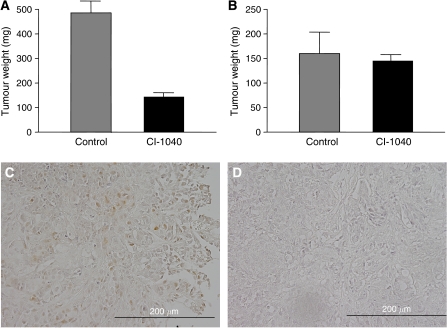
Effects of CI-1040 in a mouse xenograft model. (**A**) CI-1040-treated cells produced small tumour nodules in the peritoneal cavity. However, the diluent control-treated cells grew much larger i.p. tumours in *KRAS* mutant MDAH2774 cells. (**B**) In contrast, there were no differences in tumour weights between CI-1040-treated cells and control-treated cells in wild-type (WT) *KRAS/BRAF* SKOV3 cells. Tumours were excised and weighed. The data are expressed as the total tumour weight from each mouse. (**C** and **D**) Immunohistochemical staining of phosphorylated extracellular-regulated kinase 1/2 (p-ERK1/2) in tumours with *KRAS* mutant MDAH2774 cells. (**C**) Intense immunoreactivity is present in both the nucleus and the cytoplasm in CI-1040-untreated tumour. (**D**) Immunoreactivity is absent in both the nucleus and the cytoplasm in CI-1040-untreated tumour cells.

On the basis of the above findings, we investigated whether CI-1040 had a growth-inhibitory effect on tumour formation and development *in vivo*. Tumour xenografts from both MDAH2774 (*KRAS* mutant) and SKOV3 (wild type of *KRAS* and *BRAF*) cell lines were established in a *nu*/*nu* mouse model. All mice injected with CI-1040 developed significantly smaller intra-abdominal xenograft tumours than the mice carrying diluent control cells of the *KRAS* mutant cell line MADH2774 (Figure 7A). There were no differences in intra-abdominal xenograft tumour weights between the CI-1040-treated group and control groups transplanted with the wild-type *KRAS/BRA*F cell line SKOV3 (Figure 7B). Histological examination of the tumours after CI-1040 treatment showed inactivation of p-ERK1/2 in tumour cells based on immunohistochemistry (Figure 7C and D).

## Discussion

The significantly higher frequency of *KRAS/BRAF* mutations in non-serous type carcinomas compared with conventional high-grade serous carcinomas in this study is a finding of great interest. It suggests that conventional high-grade serous and non-serous tumours may be distinguished on the basis of characteristic genetic alterations. In addition, this observation further supports the theory that ovarian carcinoma arises from multiple pathways (Shih Ie and Kurman, 2004, 2005). In this model, conventional high-grade serous and non-serous carcinomas develop independently from one another and are characterised by different molecular genetic changes and gene expression profiles (Schwartz *et al*, 2002; Marquez *et al*, 2005).

We reported earlier that *KRAS* or *BRAF* mutations were quite common in low-grade serous ovarian carcinomas but rare in conventional high-grade serous carcinomas (Nakayama *et al*, 2006). Our present results showing low frequencies of either *KRAS* or *BRAF* mutations in conventional high-grade serous carcinoma are consistent with our earlier reports (Nakayama *et al*, 2006).

V600E is the most common *BRAF* mutation in ovarian cancer (Singer *et al*, 2003; Sieben *et al*, 2004; Shih Ie and Kurman, 2005; Nakayama *et al*, 2006). However, mutations at E585K and G463E have also been reported in ovarian cancer samples and cell lines (Davies *et al*, 2002). Therefore, further studies are needed to clarify the effects of other *BRAF* mutations in ovarian cancer, and to completely describe the mutation profile of *KRAS-BRAF* signalling in established ovarian cancer cell lines.

In this study, we also showed that the ERK–MAPK pathway was activated in 15 (33.3%) out of 45 ovarian carcinomas and activation depended on the mutational status of *KRAS* and *BRAF*. This is in contrast with a recent report showing that this pathway is frequently activated independent of the status of *KRAS* and *BRAF* in endometrioid-type endometrial cancer (Mizumoto *et al*, 2007). This discrepancy may be because of differences in organ-specific oncogenic pathways. The RAS–RAF–MEK–ERK pathway may play an important role in ovarian carcinogenesis but not in endometrial carcinogenesis. Similarly, alternative pathways for ERK activation, such as crosstalk with the PI3K pathway, exist in endometrial cancer but are rare in ovarian cancer. Indeed, PI3K signalling by either *PIK3CA* or *PTEN* mutations occurs in 40% of endometrial cancers but in <5% of ovarian cancers (Tashiro *et al*, 1997; Oda *et al*, 2005; Kolasa *et al*, 2006; Nakayama *et al*, 2006).

In this study, *KRAS* /*BRAF* mutations tended to have a favourable but not statistically significant effect on overall survival. Our findings contrast with a recent report showing a positive correlation between a *KRAS* or *BRAF* mutation and clinical aggressiveness in colorectal, non-small-cell lung, and thyroid cancers (Lievre *et al*, 2006; Lee *et al*, 2007; Massarelli *et al*, 2007). This difference in prognostic significance between ovarian cancer and the latter types is intriguing, and it probably reflects organ-specific roles of the *KRAS/BRAF* pathway. In this study, 8 out of 9 *KRAS/BRAF* mutations were identified in early stage (stage I, II) tumours. This may reflect a more indolent course of tumours with *KRAS/BRAF* mutations.

In an earlier report, advanced ovarian cancer patients (stage III, IV) with p-ERK expression had a longer overall survival than patients with low p-ERK values (Hsu *et al*, 2004). However, we did not find a significant correlation between p-ERK expression and overall survival in our study. This difference may be because of a higher percentage of early-stage ovarian cancers and endometrioid and mucinous histology tumours being included in this study as compared with the earlier study.

Although the biological roles of the RAS–RAF–MEK–ERK pathways in human cancer have been thoroughly investigated, there have been no recent studies. Therefore, it is not known whether the activation of *KRAS* or *BRAF* mutations alters the effects of these pathways on tumour progression. In this study, we carried out a genotype–phenotype correlation of ovarian cancer cells using a MEK inhibitor, CI-1040. In this study, we focussed on CI-1040 because it inhibited the common downstream target in the RAS signalling pathway. Therefore, CI-1040 has the potential to be developed into a drug for the treatment of ovarian carcinomas in patients with either *KRAS* or *BRAF* mutations. An oral formulation of CI-1040 had already been shown to be an effective MEK inhibitor and was generally well tolerated in a multicentre phase II study (Rinehart *et al*, 2004). Our results provide compelling evidence that the biological effects of the ERK signalling pathway depend on the mutational status of its upstream regulators, (i.e.), the *KRAS* and *BRAF* genes. Ovarian carcinomas with mutations in either *KRAS* or *BRAF* were more sensitive to growth inhibition and apoptosis induction by the MEK inhibitor, CI-1040. This observation suggests that ovarian carcinomas with mutations in either *KRAS* or *BRAF* are more highly dependent on the activation of the RAS–RAF–MEK–ERK pathway for cell proliferation and survival than those without such mutations. Thus, inactivation of ERK1/2 results in marked growth inhibition in ovarian carcinomas with mutations in *KRAS* or *BRAF* in comparison with only a modest effect on wild-type tumours. The above observations lend strong support to the view of ‘kinase addiction’ by which the activating mutations in the kinase pathway confer susceptibility of the tumours to an inhibitor (Sebolt-Leopold *et al*, 1999; Arteaga and Baselga, 2004). In microsatellite-unstable colorectal cancer cell lines, the effect of BRAF inhibition depended on whether the cell harboured a *BRAF* or a *KRAS* mutation. *BRAF* inhibition by small interfering RNA resulted in significantly decreased proliferation and increased apoptosis in the *BRAF* mutant lines. In contrast, this effect was not seen in the *KRAS* mutant lines (Preto *et al*, 2008). Cells carrying *BRAF* mutations have also been shown to be more sensitive to MEK inhibitors than cells with *RAS* mutations (Solit *et al*, 2006). This raises the possibility that *KRAS* and *BRAF* mutant cancer cells might be differentially dependent on signalling mechanisms that involve MEK. This difference in sensitivity to the RAS–RAF–MEK–ERK pathway between ovarian cancer and the latter types is intriguing, and it probably reflects organ-specific roles of the *KRAS and BRAF* oncogenes.

In light of our *in vivo* and *in vitro* findings, we propose that ovarian cancer patients with *KRAS* or *BRAF* mutations be considered for MEK inhibitor (CI-1040) therapy if they recur after conventional platinum and taxane chemotherapy.

Thus far, the MEK inhibitor CI-1040 has fared poorly in clinical trials for breast, colon, and lung cancer (Rinehart *et al*, 2004). However, its favourable therapeutic index and high selectivity may outweigh its shortcomings in *KRAS* and *BRAF* mutant ovarian cancer. Therefore, we recommend that in further clinical trials of MEK inhibitors for ovarian cancer, patients are stratified on the basis of *KRAS/BRAF* mutational status.

In summary, we have shown that the phenotypic change in ovarian carcinomas in response to ERK1/2 inactivation depends on the mutational status of *KRAS and BRAF*. The findings in this study provide new insight into the biological roles of the RAS–RAF–MEK–ERK signalling pathway in ovarian carcinomas. In addition, our observations have an important therapeutic implication in ovarian cancer patients with *KRAS* or *BRAF* mutations. Ovarian carcinomas with *KRAS* or *BRAF* mutation are clinically low-grade carcinomas of serous or other histological subtypes that are often refractory to conventional cytotoxic chemotherapy (Bristow *et al*, 2002a, 2002b; Winter *et al*, 2007). Therefore, detection of *KRAS* and *BRAF* mutations in ovarian cancers may identify patients who will benefit from CI-1040 therapy.

## Figures and Tables

**Table 1:** Mutational status of *KRAS* and *BRAF* genes and p-ERK1/2 expression in ovarian cancer

**Case no.**	**Histology**	**Grade**	***KRAS***	***BRAF***	**p-ERK1/2**
1	Serous	1	WT	WT	N
2	Serous	1	WT	T1796A/ V600E	P
3	Serous	2	WT	WT	N
4	Serous	3	WT	WT	N
5	Serous	3	WT	WT	N
6	Serous	2	WT	WT	P
7	Serous	2	WT	WT	N
8	Serous	2	WT	WT	P
9	Serous	3	WT	WT	N
10	Serous	3	WT	WT	P
11	Serous	3	WT	WT	P
12	Serous	3	WT	WT	N
13	Serous	3	WT	WT	N
14	Serous	3	G35T/ G12V	WT	P
15	Serous	3	WT	WT	N
16	Serous	3	WT	WT	N
17	Serous	3	WT	WT	N
18	Serous	3	WT	WT	P
19	Serous	3	WT	WT	N
20	Serous	3	WT	WT	N
21	Serous	3	WT	WT	N
22	Serous	3	WT	WT	P
23	Serous	3	WT	WT	P
24	Serous	3	WT	WT	N
25	Serous	3	WT	WT	N
26	Serous	3	WT	WT	P
27	Serous	3	WT	WT	P
28	Mucinous	1	WT	T1796A/ V600E	P
29	Mucinous	1	G35A/ G12D	T1796A/ V600E	P
30	Mucinous	1	WT	WT	N
31	Mucinous	1	WT	T1796A/ V600E	P
32	Mucinous	1	G35T/ G12V	WT	P
33	Mucinous	2	WT	WT	N
34	Mucinous	2	WT	WT	N
35	Mucinous	2	G35A/ G12D	WT	P
36	Mucinous	2	WT	WT	N
37	Mucinous	2	WT	WT	N
38	Mucinous	2	WT	WT	N
39	Mucinous	2	WT	WT	N
40	Mucinous	2	WT	WT	P
41	Mucinous	3	WT	WT	P
42	Mucinous	3	WT	WT	N
43	Mucinous	3	WT	WT	N
44	Mucinous	3	WT	WT	N
45	Mucinous	3	WT	WT	N
46	Mucinous	3	WT	WT	P
47	Mucinous	3	WT	WT	N
48	Endometrioid	1	WT	T1796A/ V600E	P
49	Endometrioid	1	WT	WT	N
50	Endometrioid	2	WT	WT	P
51	Endometrioid	2	WT	WT	N
52	Endometrioid	2	G35A/ G12D	WT	P
53	Endometrioid	2	G35A/ G12D	WT	P
54	Endometrioid	2	G35T/G12V	WT	P
55	Endometrioid	3	G35A/G12D	WT	P
56	Endometrioid	3	WT	WT	N
57	Endometrioid	3	WT	WT	P
58	Endometrioid	3	WT	WT	N

N=negative; P=positive; WT=wild type.

**Table 2:** Association between *KRAS*/*BRAF* mutational status and clinico-pathological factors in patients with ovarian cancer

		***KRAS*/*BRAF* mutation**		
**Factors**	**Patients**	**Negative**	**Positive**	***P*-value**
*FIGO stage*				
I, II	18	8	10	<0.001
III, IV	38	38	2	
				
*Grade*				
G1	9	3	6	<0.001
G2, G3	49	43	6	
				
*Histology*				
Serous	28	26	2	0.014
Others	30	20	10	
				
*Age (years)*				
<60	35	28	7	0.293
⩾60	23	17	6	
				
*p-ERK1/2*				
Positive	27	15	12	<0.001
Negative	31	31	0	

FIGO, International Federation of Gynecology and Obstetrics; MAPK, mitogen-activated protein kinase; ERKV2, Extracellular signal-regulated protein kinases1/2.
