# Supplementary material for: KRAS or BRAF mutation status is a useful predictor of sensitivity to MEK inhibition in ovarian cancer
Source: Br J Cancer. 2008 Nov 18;99(12):2020–8. doi: 10.1038/sj.bjc.6604783 (PMC2607229; doi:10.1038/sj.bjc.6604783)
Supplement: Supplementary Figures [file 6604783x1.ppt]

## Slide 1
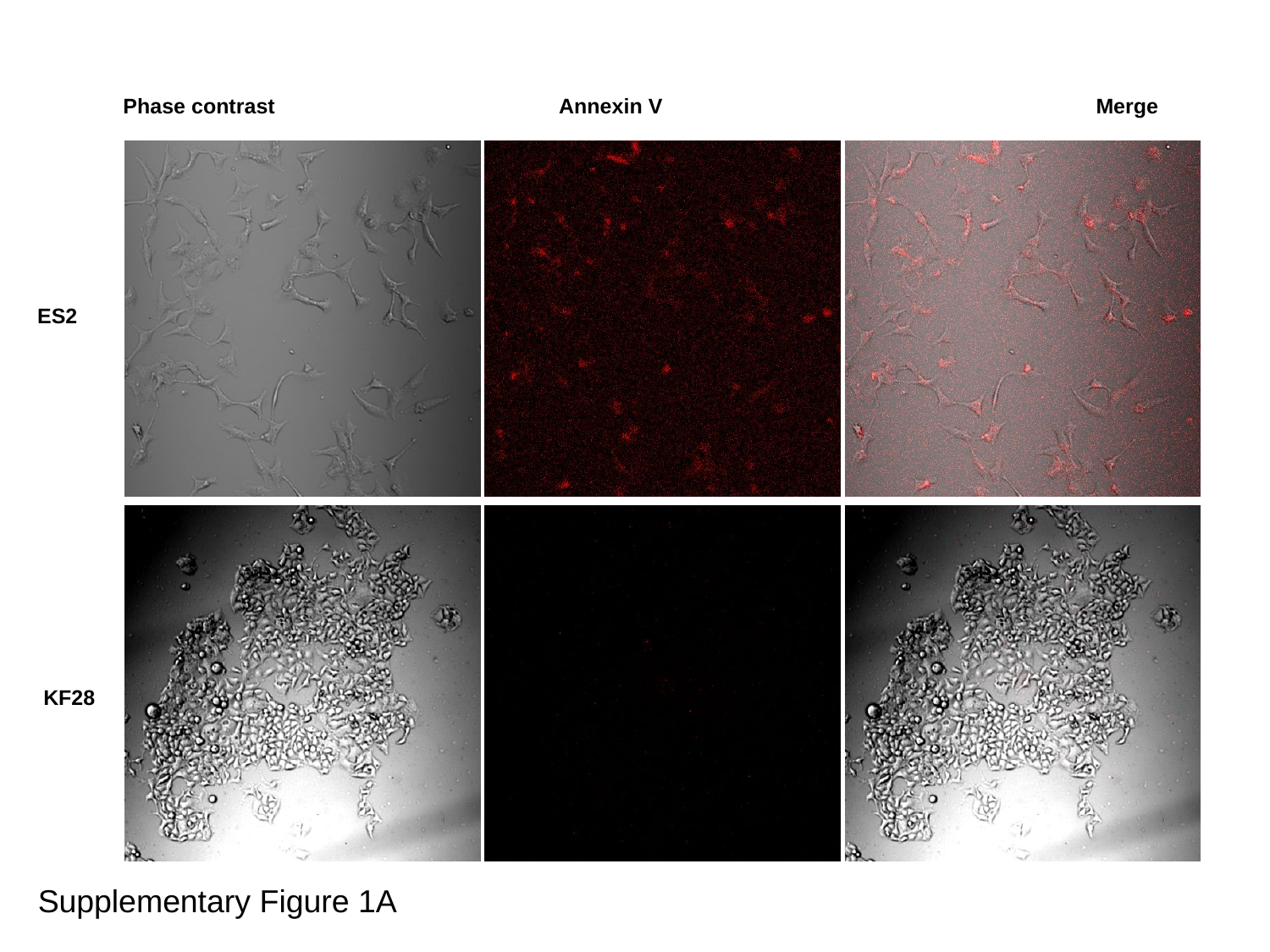

Phase contrast 　　　　　　　　　Annexin V　　　　　　　　　　　　　　　　　　　　Merge
ME180
ES2
B
A
D
C
KF28
Supplementary Figure 1A

## Slide 2
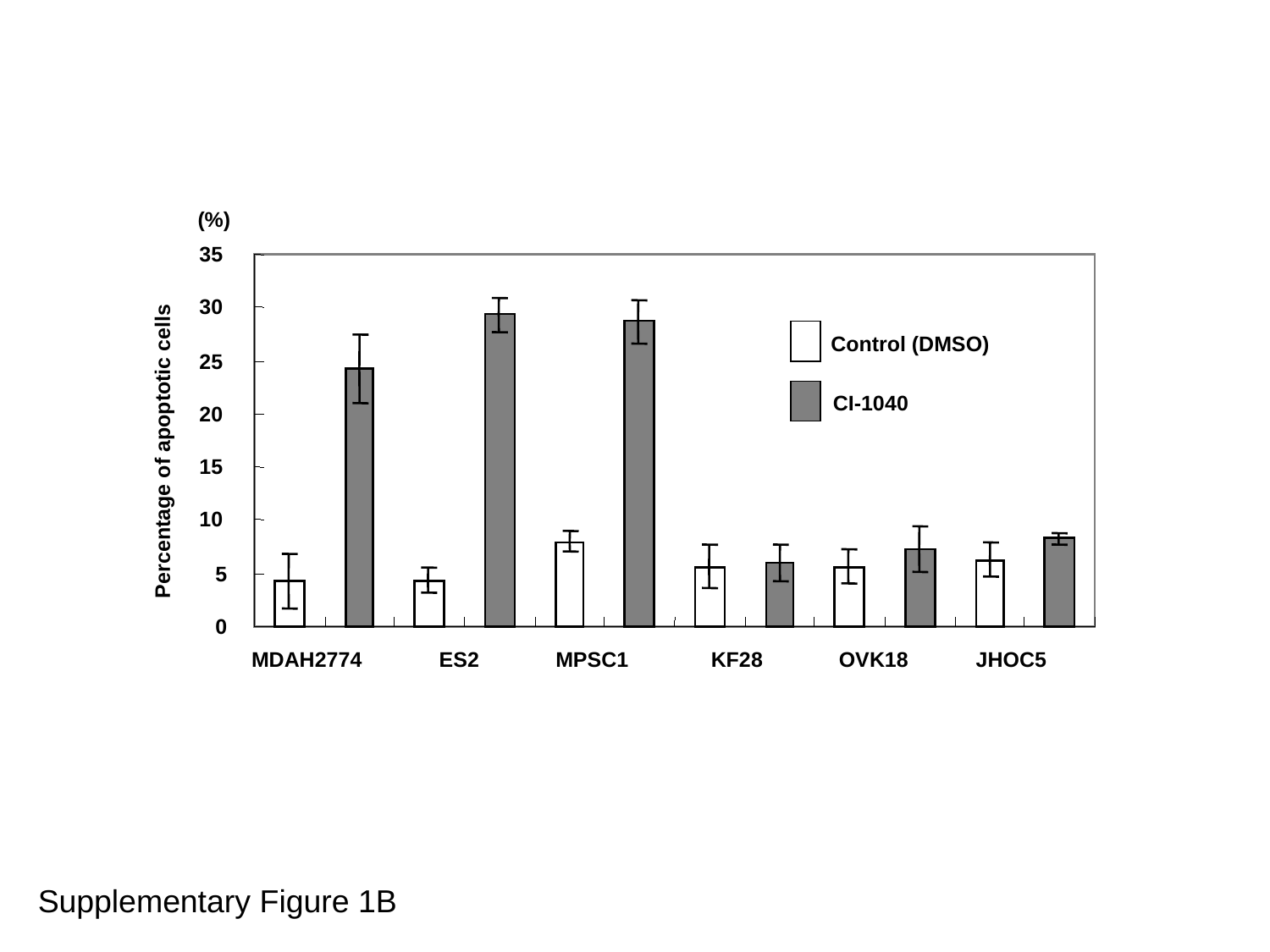

(%)
35
30
Control (DMSO)
25
CI-1040
20
Percentage of apoptotic cells
15
10
5
0
MDAH2774
ES2
MPSC1
KF28
OVK18
JHOC5
Supplementary Figure 1B
